# Neonatal Intensive Care Units Nurses’ Attitude Toward Advantages and Disadvantages of Open vs Closed Endotracheal Suction

**Published:** 2014-06-15

**Authors:** Leila Valizadeh, Raheleh Janani, Leila Janani, Fatemeh Galechi

**Affiliations:** 1 Nursing and Midwifery Faculty, Tabriz University of Medical Sciences, Tabriz, IR Iran

**Keywords:** Nurses, Infant,Newborn, Intensive Care Units,Neonatal

## Abstract

**Background:**

The vital issue of protecting the airway and maintaining ventilation in preterm infants makes tracheal suctioning an important procedure. The decision to use closed or open endotracheal suction method depends on the clinical status of infants and the nurses’ skills and preferences.

**Objectives:**

The current study aimed to compare the two methods based on the perceptions of the nurses working in Neonatal Intensive Care Units (NICU).

**Patients and Methods:**

A comparative-descriptive study carried out on 35 NICU nurses in Taleghani and Al-Zahra teaching hospitals in Tabriz, Iran, in 2013. Data were collected by self-administered questionnaire (13 Items). Data analysis, including t-test was performed using SPSS Ver. 13. A P ≤ 0.05 was considered statistically sig­nificant.

**Results:**

According to the nurses’ point of view, there are differences between characteristics of open and closed endotracheal suctioning methods (P < 0.001). By using closed endotracheal suction, the risk of traumatizing airway, developing pneumonia, increasing intracranial pressure, prolonging emergency suctioning, developing intra-ventricular hemorrhage, blood stream infection, physiological instability and lowering positive end-expiratory pressure (PEEP) are reduced. Meanwhile, lower cost, lower risk of extubation, comfort and easy washing procedure were reported as advantages of open suction.

**Conclusion:**

Closed endotracheal suctioning was evaluated to be better than the open method in the preterm neonates. More studies, especially experimental and efficient cost analysis, are recommended.

## 1. Background

Respiratory diseases are the chief reason for admission of premature neonates to NICUs (1, 2). Maintenance of breathing and patency of airway is the main objective in premature infant care (3). Most of these neonates need oxygenation and mechanical ventilation through an artificial airway like an endotracheal tube. Due to low consciousness level of neonates and weakness of their respiratory muscles, efficient removal of secretion could not happen by coughing. Therefore, intubated patients need to be suctioned (4), to prevent airway obstruction, atelectasis and pulmonary infections (5).

 Nurses are normally in charge of monitoring respiratory status and assessment of necessity for suctioning the secretions (6, 7). They must also know the new methods of suctioning (8). Endotracheal suctioning can be performed by two systems.

 The conventional way of open system is single-use and requires disconnection of the endotracheal tube from the mechanical ventilator and insertion of a suction catheter of appropriate size for the diameter of the endotracheal tube. The closed system employs a multiple-use suction catheter attached to the ventilator circuit, without disconnecting it from the patient (during suctioning) (1). During the last decade, using closed system has become more popular, but the evidence for preference of closed suction system (CSS) over open endotracheal suction (OES) has been overlooked so far (9). Use of OES or CSS depends on the clinical status of the neonates, and skill or preference of nurses (10).

## 2. Objectives

The current study aimed to compare the two methods based on the perceptions of nurses working in NICUs.

## 3. Patients and Methods

This has been a descriptive-comparative study. The study population included all nurses working in NICUs of two teaching hospitals (n = 35) during 2013. Each of them had experienced at least 10 times performing each endotracheal suctioning method. Data were collected by using a researchers’ designed questionnaire which included; demographic data, 13 statements about two kinds of suctioning method and a third section about additional advantages and disadvantages, which were not stated in the section of statements. These items were designed by using related literature, experiences and opinions of the research team. At first, 20 separate statements were written for OES and CSS.

Validity of the mentioned questionnaire was assessed by content validity method. After consulting 10 specialists (four nursing professors and six neonatologists) and applying Lawshe method, one of the statements of the provisional tool for OES and CSS was eliminated. Content validity index on the tool was 0.74. Face validity was performed under the supervision of the professors, and final format was shown on Table 1. Demographic data and the open-ended questions about additional advantages and disadvantages of OES and CSS did not change.

 Reliability of the tool was confirmed by test retest method (r = 0.87). Answers to the statements were scored from 1 to 5; 1 denotes to absolutely disagree, and 5 to quite agree. Statements number 2, 4, 5, 6, 7, 11, 12 and 13 were scored reversely. Generally, higher scores indicate better status. 

The aim of the current study was explained to the nurses. Then they were informed that completing the questionnaire as self-report and returning it were taken as the sign of participants’ consent.

### 3.1. Data Analysis

Data analysis was performed using SPSS 16 software (SPSS Inc., Chicago, IL, USA). Mean and standard deviation was calculated for quantitative variables and frequency and percentage was reported for qualitative variables. Also, t test was used. A P value less than 0.05 was considered statistically significant.

## 4. Results

All the 35 nurses were females of whom; 71.4% were from NICU of Al-Zahra teaching Hospital and 28.6% from NICU of Taleghani teaching Hospital. Of them, 88.6% of nurses were holding B.S. degree. Mean and standard deviation of nurses’ age were 34 and ± 5.35 years, also their mean working background was 9 years with the standard deviation of ± 5.35. Considering the results from nurses’ view point, closed system was generally assessed to be better. Results relating to the statements about each kind of suction system and their comparison were shown in Table 1.

**Table 1.** Comparison of Two Suction Methods (Open and Closed) in Preterm Neonates From Nurses’ View Point in NICUs ^a^, ^b^, ^c^

	Complete Agreement	Agree	No Idea	Disagree	Complete Disagree	Mean ± SD	CI %95	Tests	
								T-Test	P Value
**It is economical**								2.69	0.011
Open	5 (14.3)	11 (31.4)	5 (14.3)	8 (22.9)	6 (17.1)	3 ± 1.36	2.50-3.49		
Closed	-	6 (17.1)	5 (14.3)	8 (22.9)	16 (45.7)	2. ± 1.17	1.67-2.51		
**Performance duration is prolonged**								3.77	0.001
Open	19 (54.3)	6 (17.1)	3 (8.6)	6 (17.1)	1 (2.9)	1.93 ± 1.29	1.47-2.40		
Closed	4 (11.4)	4 (11.4)	6 (17.1)	12 (34.3)	9 (25.7)	3.56 ± 1.26	3.10-4.01		
**Extubation risk is lower**								2.57	0.015
Open	4 (11.4)	10 (28.6)	9 (25.7)	7 (20)	5 (14.3)	2.93 ± 1.2	2.48-3.38		
Closed	1 (2.9)	5 (14.3)	6 (17.1)	10 (28.6)	13 (37.1)	2. ± 1.14	1.77-2.60		
**Higher lesions in tracheal mucosa**								-7.94	
Open	19 (54.3)	7 (20)	8 (22.9)	1 (2.9)	-	1. ± 0.89	1.36-2.01		0.001
Closed	-	5 (14.3)	3 (8.6)	21 (60)	6 (17.1)	3.75 ± 0.91	3.41-4.08		
**Higher VAP probability**								-6	0.001
Open	14 (40)	12 (34.3)	6 (17.1)	3 (8.6)	-	1.93 ± 0.89	1.58-2.29		
Closed	2 (5.7)	3 (8.6)	6 (17.1)	18 (51.4)	6 (17.1)	3.62 ± 1.07	3.23-4.01		
**Higher septicemia probability**								-4.46	0.001
Open	10 (28.6)	16 (45.7)	6 (17.1)	3 (8.6)	-	2 ± 0.84	1.69-2.30		
Closed	3 (8.6)	4 (11.4)	7 (20)	18 (51.4)	3 (8.6)	3.37 ± 1.09	2.97-3.77		
**Causes change in physiologic stability**								-2.90	0.006
Open	14 (40)	8 (22.9)	7 (20)	5 (14.3)	1 (2.9)	2.18 ± 1.22	1.74-2.63		
Closed	5 (14.3)	4 (11.4)	11 (31.4)	7 (20)	8 (22.9)	3.18 ± 1.35	2.69-3.67		
**Efficient removal of secretions**								-4.73	0.001
Open	-	2 (5.7)	3 (8.6)	15 (42.9)	15 (42.9)	1. ± 0.85	1.50-2.12		
Closed	3 (8.6)	10 (28.6)	9 (25.7)	10 (28.6)	3 (8.6)	2. ± 1.12	2.56-3.37		
**Single use catheters, no self infection**								-1.08	0.28
Open	5 (14.3)	4 (11.4)	4 (11.4)	11 (31.4)	11 (31,4)	2.43 ± 1.38	1.93-2.93		
Closed	1 (2.9)	13 (37.1)	6 (17.1)	9 (25.7)	6 (17.1)	2.93 ± 1.16	2.51-3.35		
**Comfortable performance**								1.22	0.23
Open	4 (11.4)	10 (28.6)	2 (5.7)	8 (22.9)	11 (31.4)	2.62 ± 1.47	2.09-3.15		
Closed	-	6 (17.1)	6 (17.1)	11 (31.4)	12 (34.3)	2.11 ± 1.11	1.78-2.59		
**Increase in intra-cranial pressure and IVH**								-5.42	0.000
Open	14 (40)	9 (25.7)	10 (28.6)	2 (5.7)	-	1.96 ± 0.96	1.62-2.31		
Closed	1 (2.9)	2 (5.7)	15 (42.9)	12 (34.3)	5 (14.3)	3.46 ± 0.91	3.13-3.79		
**Deacreased lung volume and PEEP drop**								-3.22	0.000
Open	8 (22.9)	13 (37.1)	9 (25.7)	4 (11.4)	1 (2.9)	2.31 ± 1.02	1.94-2.68		
Closed	2 (5.7)	5 (14.3)	12 (34.3)	13 (37)	3 (8.6)	3.25 ± 1.01	2.88-3.61		
**Circuit washing is difficult**								2.67	0.011
Open	3 (8.6)	5 (14.3)	8 (22.9)	16 (45.7)	3 (8.6)	3.25 ± 1.01	2.85-3.64		
Closed	10 (28.6)	11 (31.4)	7 (20)	4 (11.4)	3 (8.6)	2.37 ± 1.26	1.91-2.83		

^a^ Abbreviation: PEEP, positive end-expiratory pressure.

^b^ Data presented as No. (%).

^c^ *d.f* = 34

Table 1 shows that according to nurses’ point of view, lesions in the tracheal mucosa, risk of pneumonia, time of performing procedure in urgency situations, increase in intracranial pressure, intra-ventricular hemorrhage (IVH) and septicemia, PEEP drop and physiological stability were all reduced applying CSS method. While, advantages of OES were reported as being economical, having less extubation risk, applying the procedure easily, and washing of the circuit.

There was a significant statistical difference between open and closed suction in about 11 statements. There was no significant statistical difference in two statements of self-infection and comfortable performance. Totally, nurses’ opinion toward mentioned 13 comments was as below; in the open suctioning method, mean ± SD was 2.31 ± 0.34 and confidence interval was 2.19-2.43, while in the closed suctioning mean ± SD was 2.99 ± 0.45 and confidence interval was 2.83-3.16, which reveals a significant statistical difference (P < 0.05).

In an answer to one of the open-ended questions, most nurses considered inverse infection as an important disadvantage of open suction. Performance of closed suction requires education and developing skills of nurses which is another disadvantage. In closed suction method, using a calibrated catheter prevents further insertion and subsequent lung injury.

## 5. Discussion

Tracheal suctioning is one of the common procedures performed by nurses in NICUs. As members of healthcare group, nurses have an important role in suctioning and their experiences on utilizing usual or new methods have great importance in the development of evidence-based practices.

 Results of the current research indicated that, nurses’ attitude toward the closed system was better. Previous studies with a similar questionnaire were not available. Nurses participating in the current study reported low lesions in the tracheal mucosa by using scaled catheters of CSS as one of the advantages of this method. In the closed suction method, using a calibrated catheter can prevent more insertion and subsequent lung injury.

Other reported advantages of CSS, were less reduction in both lung volume and PEEP drop. A major concern in relation to endotracheal suction is the reduction in lung volume, which can promote alveolar collapse; therefore, regaining the lung volume may cause acute lung injury. Arterial oxygenation is affected with the changes in lung volume (1). Studies on patients with acute lung injury showed decreased lung volume and arterial oxygenation after the use of OES. Paula et al. (1) corroborated the previous data in their study over children aged between 6 days to 13 years on whom OES and CSS were compared, and the similar results were obtained. Jongerden et al. (9) in their recent study proved that regaining lung volume in OES was longer than CSS and patients suctioned by OES exprienced more saturation drop. Thus, they recommended using CSS in infants and patients, especially those with acute lung injury who need high PEEP.

Another advantage reported in a recent study was the reduced probability of ventilator associated pneumonia. Results of the meta-analysis do not completely support the idea of reduction of pneumonia and bacterial contamination in CSS, because classification of patients and criteria of pneumonia diagnosis have been different. For different diagnostic reasons, an accurate meta-analysis study was carried out in 2007, in which; comparable criteria of pneumonia diagnosis were used but the results did not change. Thus, it seems that more studies are needed. 

Recent guidelines, recommend using CSS to prevent ventilator associated pneumonia (VAP). These recommendations are based on qualitative analysis of 3 or 4 randomized similar studies, in which it was concluded that suction system has no effect on occurrence of VAP, but despite this lack of evidence in all guidelines, the CSS method was of preference (9). Prolonged use of CSS does not increase the occurrence of VAP; therefore, it seems to be a safer procedure (10).

Findings of the present study revealed that CSS has less effect on physiological stability. Kalyn et al. in their study stated that endotracheal suction would affect physiological status of premature infants, and both systems would cause negative changes; however, in CSS, physiological stability was maintained better, and recovery time was significantly lower (10). 

Most nurses believed that OES was economical. Meta-analysis study did not provide any information about comparison of the costs, and an efficient analysis of cost seems to be necessary for both systems. It should include real costs of performing endotracheal suctioning such as material, devices, spent time by personnel, and patient outcomes during hospitalization. The duration of using CSS, which lasts from 24 hours to few days, would certainly have some effect on costs. CSS is reported more economical due to this issue that prolonged use of CSS does not increase the incidence of VAP (10).

 Nurses also believed that, the risk of extubation and replacement of the tracheal tube in OES was lower. In CSS, this risk can be due to the vacuum created in the circuit, which can be overcome by minimizing suctioning time and adjusting suction pressure. Necessity of supporting tracheal tube during suctioning should be noted, otherwise in both methods extubation might occur.

In conclusion, NICU nurses reported that CSS was a better method for premature infants. Advantage of CSS was a reduction in all of these complications: lesions in the tracheal mucosa, pneumonia probability, time of procedure in urgency situations, increase in intracranial pressure, IVH, septicemia, PEEP drop and physiological instability. While, the low costs, less extubation risk, simplicity of procedure, and easy cleaning of the circuit were the advantages of OES.

 This study was descriptive and reflected nurses’ attitudes. Sample size was restricted. Finally, we suggest that more cost analysis and experimental studies about CSS over premature infants be designed and carried out.

## References

[1] Paula LC, Ceccon ME (2010). Randomized, comparative analysis between two tracheal suction systems in neonates.. Rev Assoc Med Bras..

[2] Holmstrom ST, Phibbs CS (2009). Regionalization and mortality in neonatal intensive care.. Pediatr Clin North Am..

[3] Sheikh-bahhaeddin-zadeh E, Raee V (2012). NICU nursing..

[4] Arezomanias S (2003). Newborn Nursing..

[5] Verklan MT, Walden M (2010). Core curriculum for neonatal intensive care nursing..

[6] William SC, Asquith JMF (2000). Pediatric intensive care nursing..

[7] James SR, Ashwill JW, Droske SC (2007). Nursing care of children: principles & practice..

[8] Khamis GM, Waziry OG, Badr-El-Din AHA, El-Sayed MM (2011). Effect of Closed Versus Open Suction System on Cardiopulmonary Parameters of Ventilated Neonates.. J Am Sci..

[9] Jongerden IP, Rovers MM, Grypdonck MH, Bonten MJ (2007). Open and closed endotracheal suction systems in mechanically ventilated intensive care patients: a meta-analysis.. Crit Care Med..

[10] Kalyn A, Blatz S, Sandra F, Paes B, Bautista C (2003). Closed suctioning of intubated neonates maintains better physiologic stability: a randomized trial.. J Perinatol..

